# Sentiment analysis and causal learning of COVID-19 tweets prior to the rollout of vaccines

**DOI:** 10.1371/journal.pone.0277878

**Published:** 2023-02-24

**Authors:** Qihuang Zhang, Grace Y. Yi, Li-Pang Chen, Wenqing He

**Affiliations:** 1 Department of Statistical and Actuarial Sciences, University of Western Ontario, London, Ontario, Canada; 2 Department of Computer Science, University of Western Ontario, London, Ontario, Canada; 3 Department of Epidemiology and Biostatistics, McGill University, Montreal, Quebec, Canada; 4 Department of Statistics, National Chengchi University, Taipei, Taiwan; Zayed University, UNITED ARAB EMIRATES

## Abstract

While the impact of the COVID-19 pandemic has been widely studied, relatively fewer discussions about the sentimental reaction of the public are available. In this article, we scrape COVID-19 related tweets on the microblogging platform, Twitter, and examine the tweets from February 24, 2020 to October 14, 2020 in four Canadian cities (Toronto, Montreal, Vancouver, and Calgary) and four U.S. cities (New York, Los Angeles, Chicago, and Seattle). Applying the RoBERTa, Vader and NRC approaches, we evaluate sentiment intensity scores and visualize the results over different periods of the pandemic. Sentiment scores for the tweets concerning three anti-epidemic measures, “masks”, “vaccine”, and “lockdown”, are computed for comparison. We explore possible causal relationships among the variables concerning tweet activities and sentiment scores of COVID-19 related tweets by integrating the echo state network method with convergent cross-mapping. Our analyses show that public sentiments about COVID-19 vary from time to time and from place to place, and are different with respect to anti-epidemic measures of “masks”, “vaccines”, and “lockdown”. Evidence of the causal relationship is revealed for the examined variables, assuming the suggested model is feasible.

## 1 Introduction

The COVID-19 disease, caused by the human severe acute respiratory syndrome coronavirus 2 (SARS-Cov-2), was declared to be a pandemic by the World Health Organization (WHO) in March of 2020. This disease has caused over 500 million infections and 6 million deaths all over the world as of June 1, 2022 [1]. While extensive studies have been conducted to examine the impacts of COVID-19 on public health, investigations of the impact on people’s emotions are relatively limited.

**Fig 1:**
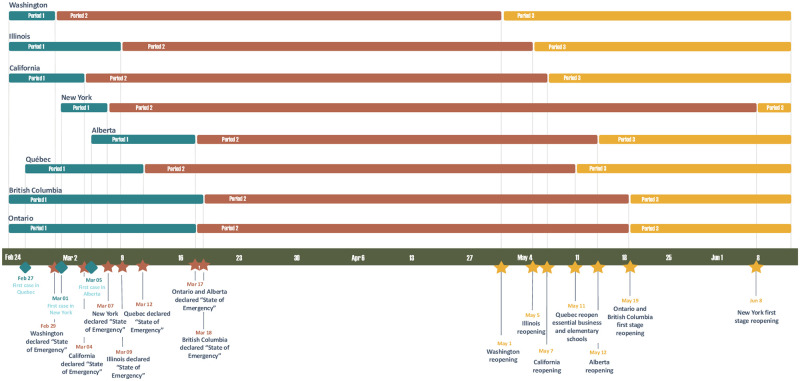
The timeline of the measures, indicated by the three periods, which are taken by the four provincial governments in Canada: Alberta, Quebec, British Columbia, and Ontario, and four state governments in U.S.: Washington, Illinois, California, and New York.

The first case of COVID-19 in Canada was reported in Toronto on January 23, 2020 [2]. To prevent the spread of COVID-19, the four most populated provinces in Canada consecutively announced the “state of emergency” (displayed in Fig 1), taking the measures of shutting down public business, banning social gatherings, imposing social distancing, and requiring masks-wearing in the public area, etc. As the situation of disease spreading ameliorated during the period from July to August of 2020, the “state of emergency” was relaxed to various extents in different provinces. With the roaring number of newly infected cases, the “state of emergency” was subsequently restored again in all the four provinces [3].

The COVID-19 pandemic has become one of the most discussed topics on the internet since it was first reported in January 2020 [4]. For example, as one of the most widely used social media platforms, Twitter has attracted extensive attention from the public to share their opinions and feelings about COVID-19. To extract valuable messages, it is of interest to conduct text mining of the public information posted on Twitter. Text mining is a commonly used technique to explore corpus; for common strategies of analyzing sentiments, see Chapter 4 of Kwartler [5].

Studies about the public emotional reaction to the COVID-19 pandemic have been available by applying text mining techniques to analyze Twitter data. For example, Xue et al. [6] implemented the Latent Dirichlet Allocation (LDA) method to categorize the tweets by topics and compared their sentiments. Khanday et al. [7] extracted relevant features and then classified textual clinical reports using machine learning algorithms. Most available texting mining work about COVID-19 data (e.g., [8–10]), however, focuses on exploratory analysis using visualization techniques such as word clouds and histograms to summarize the most-posted words.

It is interesting to conduct more refined analyses to assess how the public sentiments may change dynamically with the evolvement of the pandemic and how they may be associated with different measures implemented by the governments to mitigate the virus spread. In this article, we provide new studies to enhance the understanding of the impact of COVID-19 on public sentiments. Our research serves as an addition to the existing work about COVID-19. In particular, we carry out two studies to dynamically explore Twitter data to assess public sentiments about the COVID-19 pandemic.

First, we conduct sentiment analysis to characterize the topic popularity and public sentiments about COVID-19. Then, we compare the sentiment scores for different regions dynamically with temporal effects incorporated. We are also interested in people’s reactions related to the keywords “lockdown”, “masks”, and “vaccine” in the analysis. In the second analysis, we build a statistical model to examine the relationship among sentiment scores and Twitter activities, such as “tweet”, “like”, “reply” and “retweet”. We are interested in studying how the public reactions collected on Twitter may be associated with the tweet activities and whether any possible causal relationship may be found.

While identifying causal relationships from observational time series is challenging, various methods have been proposed, including the Granger method and convergent cross mapping (CCM). Granger [11] initiated the following idea for examining the causal relationships for time series data. If time series {*Y*_*t*_ : *t* = 1, 2, …} causes time series {*X*_*t*_ : *t* = 1, 2, …}, then the value *Y*_*t*_ at current time *t* should be able to improve the prediction of *X*_*s*_ for future times *s* > *t*. Alternatively, Takens [12] viewed causality from a different perspective. If {*Y*_*t*_ : *t* = 1, 2, …} causes {*X*_*t*_ : *t* = 1, 2, …}, then the current value *X*_*t*_ can be used to reconstruct the history of *Y*_*s*_ with *s* < *t*. The Granger causality model imposes the linear assumption for the relationship between {*Y*_*t*_ : *t* = 1, 2, …} and {*X*_*t*_ : *t* = 1, 2, …}, which may perform poorly for nonlinear dynamical systems. As a remedy, the CCM method [13] was developed to deal with nonlinear systems. Further, Huang et. al. [14] developed an extension of the CCM method (ECCM) to accommodate the time-lagged statistics using the “Reservoir Computing”, a computationally efficient procedure for handling nonlinear dynamical systems. In our second analysis, we employ the ECCM method to reflect the nonlinear dynamic nature of the COVID-19 data [15].

To alleviate possible confounding effects associated with underlying factors including culture, government practice, internet access regulations, etc., here we restrict our attention to the four most populated cities in Canada, (i.e., Toronto, Montreal, Vancouver, and Calgary), and the three most populated cities in the United States, (i.e., New York, Los Angeles, and Chicago), together with Seattle which is in the vicinity of Vancouver. Our analyses are conducted on the data arising from Twitter for the period from February 24, 2020 to October 14, 2020, a period prior to the rollout of the COVID-19 vaccines in North America.

The remainder of the article is organized as follows. In Section 2, we first present the procedure of obtaining tweets from Twitter, and in Section 3, we describe the methods for sentiment analysis and causal inference about time series data. In Section 4, we carry out two analyses and compare the results over different periods. We conclude the article with discussions presented in the last section.

## 2 Data processing

Our Twitter data mining pipeline consists of data preparation and analysis stages, where the data preparation procedure includes data collection and cleaning. The clean data are then analyzed using the **pandas** package of Python3.8. The data analysis stage includes the construction of descriptive statistics characterizing the topic popularity and public sentiments. We compare the sentiment scores within different regions dynamically with temporal effects incorporated. The details are described in the sequel.

### 2.1 Data collection

COVID-19 related tweets, published between February 24, 2020 and October 14, 2020, are collected from four Canadian cities: Toronto, Montreal, Vancouver, and Calgary, the representative cities of the four most populated provinces, Ontario, Quebec, British Columbia, and Alberta, respectively. Four cities in the U.S.: New York, Los Angeles, Seattle, and Chicago, are considered for comparison.

We use the **snscrape** module (https://github.com/JustAnotherArchivist/snscrape) of Python3.8 to scrape those tweets having “COVID-19” as keywords for each considered city, where we consider the area within 50*km* of the center of each city for simplicity. This leads to 30,655 tweets in total for the four cities in Canada and 69,742 tweets in total for the four cities in the United States during the study period.

### 2.2 Data cleaning

Applying the common standards (e.g., [6, 16]), we first clean raw data using the **pandas** module of Python3.8, as in [6], to remove the following symbols from the tweets in the data set: (1) URLs, hashtag symbol “#”, and symbol “@”; and (2) Meaningless characters, punctuation, and stop-words. Tweets written in a non-English language are also removed. Table 1 displays some examples of meaningless characters, punctuation, and stop-words.

**Table 1:** An example list of meaningless characters, punctuation, and stop-words.

Character	Punctuation	Stop-words
**	,	I
SSS	()	me
=	;	myself
$	-	during
&	∼	before

## 3 Analysis methods

### 3.1 Sentiment analysis

To reflect how tweets are connected with moods and sentiments, we carry out sentiment analysis which is a process of revealing authors’ attitudes about the discussed topic from the text. We consider two types of commonly used methods for evaluating text sentiments: bag-of-words based methods and natural language processing techniques.

#### 3.1.1 Bag-of-words based methods

Bag-of-words based methods break each tweet into individual words, determine the sentiment score of each word in the tweet according to a lexicon, and then obtain the sentiment score for each tweet by summing up those scores of the words.

Different lexicons have been proposed for text mining. Some lexicons classify a sentiment as *negative*, *neutral*, or *positive* (e.g., [17]). The Bing [18] lexicon classifies words in a binary manner, either positive or negative. The Valence Aware Dictionary for sEntiment Reasoning (Vader), proposed by Gilbert and Hutto [19], takes the intensity of the sentiment into account, where the sentiment intensity score is a scale ranging between negative and positive values. National Research Council (NRC) Sentiment and Emotion Lexicons [20] consider each word to be one or a combination of multiple moods, including *anticipation*, *positive*, *negative*, *sadness*, *disgust*, *joy*, *anger*, *surprise*, *fear*, and *trust*. Examples of those lexicons are given in Table 2.

**Table 2:** Examples of different lexicons: Vader, NRC and Bing.

Vader		NRC		Bing	
word	score	word	sentiment	word	sentiment
:-)	1.3	abacus	trust	abominable	negative
lmao	2.0	abandon	fear	abominably	negative
lol	2.9	abandon	negative	abominate	negative
abducted	-2.3	abandon	sadness	abomination	negative
abduction	-2.8	abandoned	anger	abort	negative
agrees	1.5	accident	negative	admonition	negative
alarm	-1.4	accident	sadness	adorable	positive
alarmed	-1.4	accident	surprise	adore	positive
alarmist	-1.1	accidental	fear	adored	positive
amaze	2.5	accidental	negative	adorer	positive
amort	-2.1	accidental	surprise	adoring	positive

Here, we use Vader and NRC, respectively, to characterize the sentiment and emotion of tweets. The Vader lexicon quantifies the sentiment into numeric scores which can be used for further analysis, and the NRC lexicon provides detailed categories to describe the mood in a refined fashion. The Vader lexicon is also specifically attuned to sentiments expressed in social media. It contains the utf-8 encoded emojis and emoticons, which are important features frequently used in tweet texts [19].

To conduct sentiment analysis, polarity scores are identified for every single word in each tweet according to the Vader lexicon, and the frequency of each mood in emotion categories appearing in the text is identified according to the NRC lexicon. For the sentiment score obtained by Vader, we calculate the average sentiment score of each tweet by first summing up the scores of the words in the tweet and then dividing by the total number of words in the tweet. Following Gilbert and Hutto [19], after calculating sentiment scores, we further adjust the calculated tweet-wise sentiment scores to incorporate the information related to negation words (“not” and “n’t”), punctuation to intensify sentiments (e.g., “Good!!!”), conventional use of word-shape to signal emphasis (e.g., using CAPITAL words), words modifying the intensity (e.g., “very”, “pretty”, etc.), and conventional slangs and emoji (e.g, “lol”,“:)”). For the case with “??”, Vader treat it as an intensified punctuation just like “!!”, but for the case with “?”, no adjustment is made because of the uncertainty of differentiating between real question and a rhetorical question. After computing positive, neutral, and negative scores for each tweet, we further calculate the compound score according to the rules of Gilbert and Hutto [19], and then normalize it to be between -1 (most negative) and +1 (most positive). These steps are implemented via the polarity_scores() function in the **Vader** module (https://github.com/cjhutto/vaderSentiment).

For the mood categories annotated by the NRC lexicon, the accumulating number of counts of each emotion category is also calculated for each tweet. We further compute the frequency, defined as the ratio of the counts of emotions to the total word count in a tweet, using the NRClex() function in the python **NRCLex** module (https://github.com/metalcorebear/NRCLex). For each word, the emotion is represented by a ten-dimensional vector to reflect 10 different moods specified for NRC, where each element is expressed as the frequency of a mood, ranging from 0 (extremely lack of that emotion) to 1 (extremely full of that emotion).

#### 3.1.2 Natural language processing based methods

In contrast to bag-of-words based methods discussed in Section 3.1.1, we employ *Robustly Optimized BERT Pretraining Approach* (RoBERTa) [21] to characterize sentiments and emotions of tweets. RoBERTa is an improved pretrain model of *Bidirectional Encoder Representations from Transformers* (BERT) in that it has longer training epochs, bigger training batches, and more training data. BERT [22] is a useful technique for natural language processing (NLP) that origins from pre-training contextual representations. It applies the bidirectional training of Transformer [23] to learn contextual relationships among words in a text. This technique is used to pretrain deep bidirectional representations, expressed as numerical vectors, to show the meaning of words from the text using *multi-layer bidirectional Transformer* [23]. It takes an input of a text sentence and produces an output of a 3-dimensional vector of sentiment scores, with the three components corresponding to the probability of positive, neutral, and negative sentiments. Then, we calculate the RoBERTa sentiment scores as the probability difference for the positive and negative sentiments.

We implement *Twitter-RoBERTa-base*, which is pretrained based on Twitter data with over 58 millions tweets. In the implementation, we use python module *transformer* to apply the pretrained model downloaded from an online machine learning platform (https://huggingface.co/cardiffnlp/twitter-roberta-base-sentiment) to assess sentiment scores for tweets.

As an example, we consider five COVID-19 related tweets in Toronto posted on February 24 of 2020 that are listed in Table 3, and we calculate their sentiment scores using the RoBERTa and bag-of-words methods. The resulting sentiment scores are respectively presented in Tables 3 and 4.

**Table 3:** Examples: Five COVID-19 related tweets collected in Toronto on February 24 of 2020 and their sentiment scores calculated using RoBERTa.

		RoBERTa			
No.	Tweet	negative	neutral	positive	score
1	The trajectory of the coronavirus is unknown at this time and it’s possible that cases have occurred in other countries that don’t have the proper tools to diagnose and contain it.	0.713	0.272	0.015	-0.698
2	GIS real-time data map of coronovirus COVID19 from john hopkins	0.050	0.874	0.076	0.026
3	Fantastic thread by our Dr. Hota describing how the system works to protect Canadians	0.006	0.159	0.835	0.829
4	…and flights are still letting people in?	0.626	0.350	0.024	0.626
5	passenger on montreal to vancouver aircanada flight tested positive for coronavirus COVID-19	0.346	0.597	0.058	-0.288

**Table 4:** Examples: Sentiment scores of the five COVID-19 related tweets in Table 3 calculated using Vader and NRC.

	Vader				NRC									
No.	Negative	Neutral	Positive	Compound	Anticipation	Positive	Negative	Sadness	Disgust	Joy	Anger	Surprise	Fear	Trust
1	0	1	0	0	9	8	8	8	8	7	8	8	8	7
2	0	1	0	0	2	2	3	1	3	1	2	1	2	2
3	0	0.721	0.279	0.7351	3	4	5	4	2	3	3	2	3	4
4	0	1	0	0	3	4	4	4	4	4	4	3	4	3
5	0	0.769	0.231	0.5574	2	2	2	2	2	2	2	2	2	3

### 3.2 Causal inference

#### 3.2.1 Examination framework

With the descriptive analysis about sentiment scores reported in Section 3.1, we are further interested in examining potential causal relationships for paired variables concerning COVID-19 tweet activities and sentiment scores.

Let *X* and *Y* denote two of the examined variables. We are interested in identifying whether there is evidence to suggest a causal relationship between *X* and *Y* by examining {*X*_*t*_ : *t* = 1, …, *T*} and {*Y*_*t*_ : *t* = 1, …, *T*}, the time series of observations for *X* and *Y*, respectively, where *X*_*t*_ and *Y*_*t*_ are observed values of *X* and *Y* at time *t*, respectively, and *T* is the end of the study. To this end, we implement convergent cross mapping [24], integrated with the echo state network approach [25], to explore pairwise causal relationships.

The idea of the convergent cross mapping is that if *Y* is the cause of *X*, then the time series {*Y*_*t*_ : *t* = 1, …, *T*} of the causal variable *Y* can be recovered from the time series {*X*_*t*_ : *t* = 1, …, *T*} of the effect variable *X* [26]. To facilitate this rationale and possible lag effects, let *τ* denote the lag time, and we take *X*_*t*_ as the input data and repeatedly fit an leaky echo state network model [27] to predict *Y*_*t*+ *τ*_ by varying the value of *τ*, denoted ${\hat{Y}}_{t+\tau }$. The detail is given in Section 3.2.2.

Now we evaluate the performance of the prediction of *Y*_*t*+*τ*_. For any given *τ* ≤ *T*, we compute the Pearson correlation coefficient between the predicted time series ${\hat{Y}}_{t+\tau }$ and their corresponding observations *Y*_*t*+*τ*_ [24], denoted *ρ*_*y*_(*τ*) and given by

$$\begin{array}{c} \rho_{y}\left(\tau \right)=\frac{\sum_{t=1+|\tau |-h(\tau )}^{T-h(\tau )}Y_{t+\tau }{\hat{Y}}_{t+\tau }-(\sum_{t=1+|\tau |-h(\tau )}^{T-h(\tau )}Y_{t+\tau })(\sum_{t=1+|\tau |-h(\tau )}^{T-h(\tau )}{\hat{Y}}_{t+\tau })}{\sqrt{\sum_{t=1+|\tau |-h(\tau )}^{T-h(\tau )}{\left(Y_{t+\tau }-{\bar{Y}}_{t+\tau }\right)}^{2}\times \sum_{t=1+|\tau |-h(\tau )}^{T-h(\tau )}{\left({\hat{Y}}_{t+\tau }-{\bar{\hat{Y}}}_{t+\tau }\right)}^{2}}}, \end{array}$$ (1)

where ${\bar{Y}}_{t+\tau }=\frac{1}{T-|\tau |}\sum_{t=1+|\tau |-h(\tau )}^{T-h(\tau )}Y_{t+\tau }$, ${\bar{\hat{Y}}}_{t+\tau }=\frac{1}{T-|\tau |}\sum_{t=1+|\tau |-h(\tau )}^{T-h(\tau )}{\hat{Y}}_{t+\tau }$, and

$$\begin{array}{c} h\left(\tau \right)=\{\begin{array}{cc} \tau , & \text{if}\;\tau \geq 0,\\ 0, & \text{if}\;\tau <0. \end{array} \end{array}$$ (2)

Likewise, if *X* is the cause of *Y*, the procedure described above is repeated by swapping *X* and *Y*. That is, taking *Y*_*t*_ as the input data and *X*_*t*+*τ*_ as output labels with *τ* denoting the lag time, we fit an echo state network model to predict the values of *X*_*t*+*τ*_, denoted as ${\hat{X}}_{t+\tau }$. Then, for any given *τ*, we compute the Pearson correlation coefficient between ${\hat{X}}_{t+\tau }$ and *X*_*t*+*τ*_, denoted as *ρ*_*x*_(*τ*), in the same manner as (1).

Following the lines of [14, 24], we determine the causality between X and Y according to the time lag *τ* so that *ρ*_*x*_(*τ*) and *ρ*_*y*_(*τ*) reach their peak values. Specifically,

- If *X* causes *Y* and not vice versa, we expect the peak value of the *ρ*_*x*_(*τ*) to be located in the positive domain, i.e., the corresponding *τ* is positive. Meanwhile, we expect the peak value of the *ρ*_*y*_(*τ*) to be at a negative *τ*.
- If *X* and *Y* cause each other, both the peak values of the *ρ*_*x*_(*τ*) and the *ρ*_*y*_(*τ*) are expected to occur in the negative domain.
- If the coupling of *X* and *Y* has a delay effect, the lag positions of the peaks of the *ρ*_*x*_(*τ*) and the *ρ*_*y*_(*τ*) are expected to be influenced by the delay time.

To study how *ρ*_*x*_(*τ*) and *ρ*_*y*_(*τ*) may change at different time lag *τ*, we consider the range [−30, 30] for *τ* in the analysis.

#### 3.2.2 Echo state network

As discussed in Section 3.2.1, the identification of the causality relationship of *Y* leading to *X* is carried out by examining the Pearson correlation *ρ*_*y*_(*τ*), which is obtained by fitting a prediction model connecting *X* and *Y*. While various modeling schemes may be considered, here we employ the echo state network approach for its good performance in the prediction of non-linear time series data [25].

**Fig 2:**
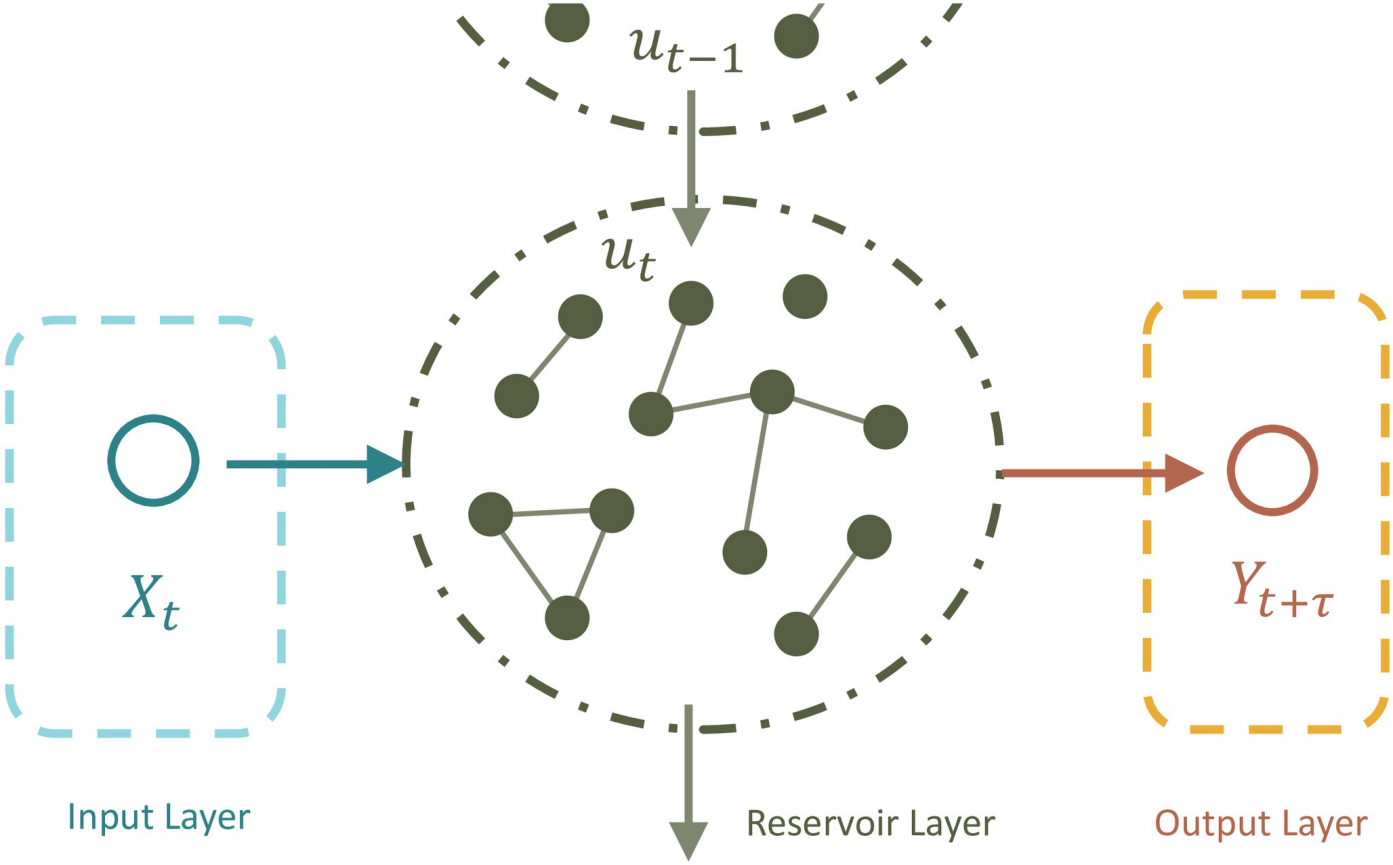
The diagram of an echo state network.

The echo state network is basically composed of three layers: the input layer, the reservoir layer, and the output layer, as shown in Fig 2. The input layer contains the input data (*X*_*t*_) and the output layer is made up of the output labels (*Y*_*t*+*τ*_). The reservoir layer consists of the hidden reservoir neuron states, denoted *u*_*t*_, which form an *N* × 1 vector with *N* being a user-specified positive integer. An *N* × *N* adjacent matrix, say *A*, is constructed to describe the connections among the reservoir neurons.

The determination of the links among the three types of layers is carried out with two procedures: the input-to-reservoir and the reservoir-to-output procedures, described as follows:

- From the input layer to the reservoir layer:
  For any *t* = 1, …, *T*, we construct the relationships between the input data *X*_*t*_ and the layers of hidden reservoir neuron states. Let *u*_0_ denote the initial reservoir neuron states, then we update the neuron states *u*_*t*_ by
  $$\begin{array}{cc} u_{t}^{*} & =\text{tanh}\{Au_{t-1}+W_{\text{in}}X_{t}\};\\ u_{t} & =\left(1-\psi \right)u_{t-1}+\psi u_{t}^{*}, \end{array}$$
  where *ψ* is the leaky parameter to be specified, and *W*_in_ is an *N* × 1 matrix representing the weight to transform *X*_*t*_ from input layer to reservoir layer. Here, $\text{tanh}\left(v\right)=\frac{\text{exp}(v)-\text{exp}(-v)}{\text{exp}(v)+\text{exp}(-v)}$.
- From reservoir layer to output layer:
  We compute the predicted output labels,
  $$\begin{array}{c} {\hat{Y}}_{t+\tau }=W_{\text{out}}u_{t}, \end{array}$$
  where *W*_out_ is an 1 × *N* matrix representing the weight matrix transform *u*_*t*_ into output layer.

In these steps, only the weights *W*_out_ need to be trained by minimizing the penalized loss function

$$\begin{array}{c} {\left\|{\hat{Y}}_{t+\tau }-Y_{t+\tau }\right\|}_{2}+\alpha {\left\|W_{\text{out}}\right\|}_{2}, \end{array}$$

where ‖⋅‖_2_ represents the *L*_2_-norm and *α* is the tuning parameter to be specified.

The adjacent matrix *A* and the weight matrix *W*_in_ are prespecified using the following procedure. Let *p*_*s*_ be a user-specified value between 0 and 1, and let *γ* be a user-determined positive scaling parameter determining the degree of nonlinearity in the reservoir dynamics [28]; and let *β* be a pre-specified positive scaling parameter. Generating a sequence of values, say *v*, independently from Uniform [−1, 1], and a sequence of values, say *s*, independently from Bernoulli(*p*_*s*_), we form the weight matrix *W*_in_ by letting its elements be given by *γsv*. The adjacent matrix *A* is formed similarly with its element given by *βsv*. To ensure the echo state network to work properly, the effects of initial conditions should vanish as the times series evolves [29], which is also known as the echo state property. A necessary condition to ensure the echo state property is that the largest eigenvalue of *A*, denoted λ_max_, is smaller than 1 [27]. Here, λ_max_ determines the time range that the time series data interact each other non-linearly. Such a property guides us to set a suitable value for scale parameter *β*.

Treating variable *X*_*t*_ as the input data and *Y*_*t*+*τ*_ as the output labels, we implement the echo state network using the EchoTorch module [30] in Python3.8. To determine suitable values of the tuning parameters λ_max_, *ψ*, *N*, *p*_*s*_, *α*, and *γ*, we take the leave-one-out cross-validation procedure. Specifically, in our data analysis in Section 4, we take the time series of seven cities as the training data and the remaining one city as the testing data, and this procedure is repeated by taking each city as testing data once, where in each study, we record the normalized root-mean-squared-error (NRMSE) [25]:

$$\begin{array}{c} \text{NRMSE}=\frac{\sqrt{\sum_{t=1}^{T}{\left({\hat{Y}}_{t}-Y_{t}\right)}^{2}/T}}{\sum_{t=1}^{T}Y_{t}/T}. \end{array}$$

We conduct a grid search to find the optimal set of tuning parameters such that the NRMSE is minimized. Table 5 displays the grids to be searched for the associated parameters for the analysis to be conducted in Section 4.

**Table 5:** Values for the parameters in the grid search.

Parameter	Grid Search Values
spectral radius λ_max_	0.1, 0.3, 0.5, 0.7, 0.9
reservoir size *N*	50, 100, 150, 200, 250, 300
connectivity *p*_*s*_	0.1, 0.6
ridge parameter *α*	10^−10^, 10^−7^, 10^−4^, 0.1, 100
input scaling *γ*	0.1, 0.6

## 4 Data analysis results

### 4.1 Descriptive statistics

**Fig 3:**
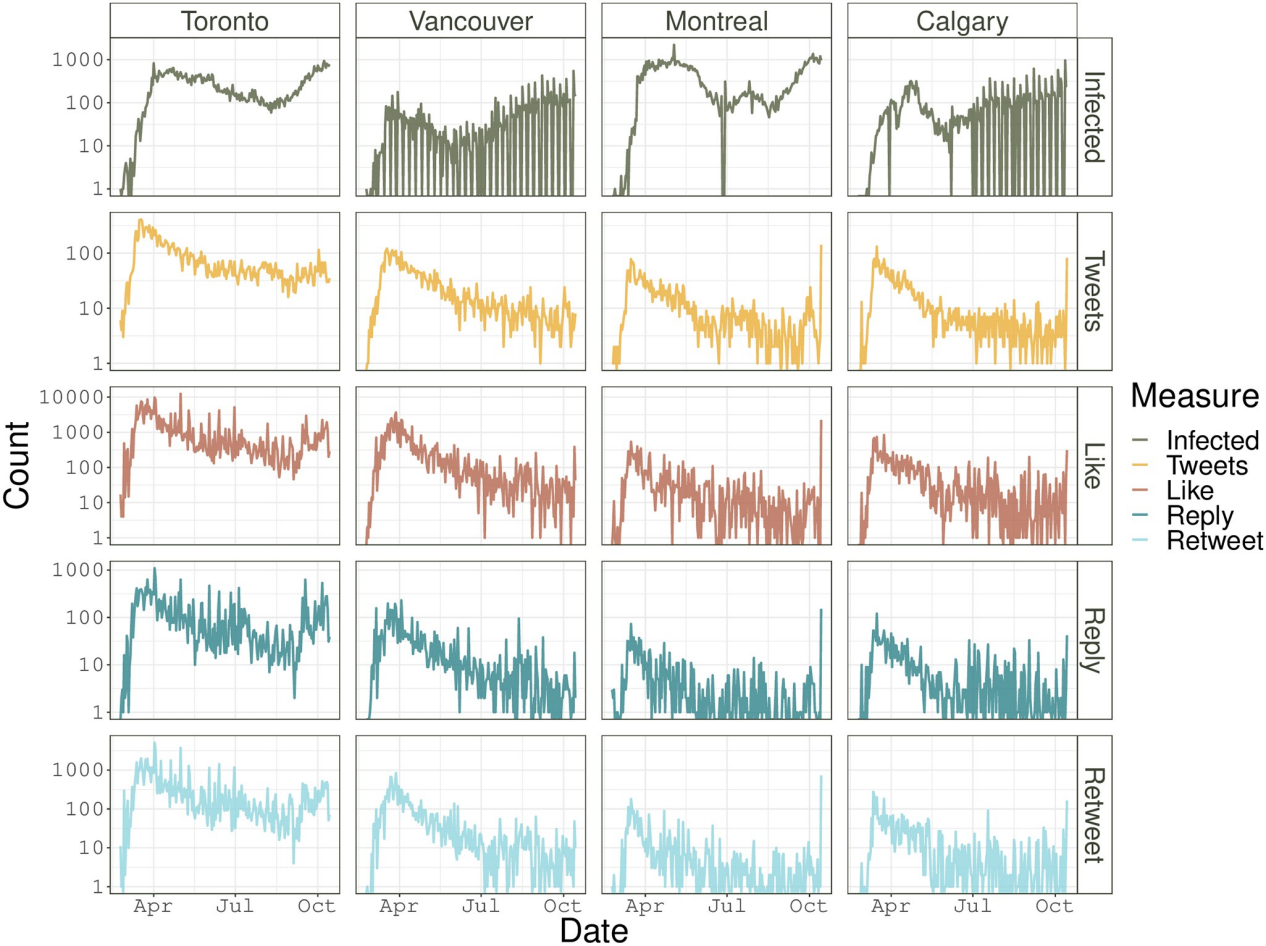
Canadian data: The trajectory of provincial daily infected cases, daily number of tweets, total counts of likes, reply and retweets of the COVID-19 related tweets for Toronto, Vancouver, Montreal, and Calgary. The y-axis is presented on the scale of logarithm.

**Fig 4:**
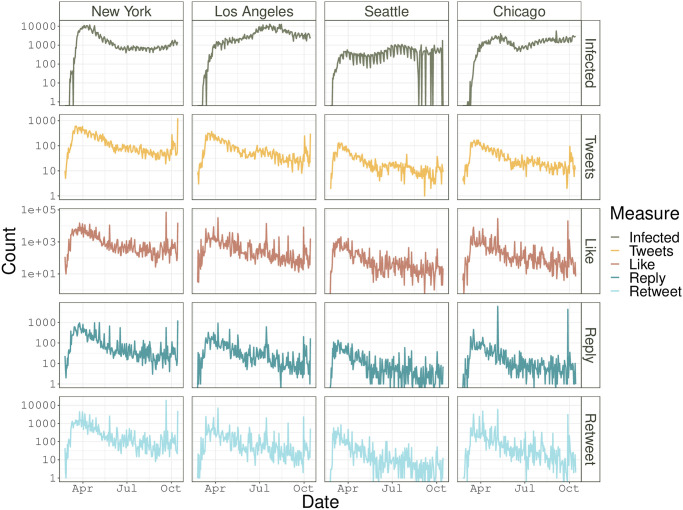
U.S. data: The trajectory of state daily infected cases, the daily number of tweets, total counts of likes, replies, and retweets of the COVID-19 related tweets for New York, Los Angeles, Seattle, and Chicago. The y-axis is presented on the scale of the logarithm.

Before conducting text mining of tweets, we summarize demographic information of the tweets data scraped online. The number of tweets may reflect the popularity of a topic on Twitter; and “like”, “reply” and “retweet” are three main activities for users to engage with the tweet and their counts indicate the impact of a tweet in generating discussions. Figs 3 and 4 present the results for the cities in Canada and the U.S., respectively, where we report the city-level trajectory of the number of tweets and of the total number for each of “like”, “reply”, and “retweet” for COVID-19 associated tweets, in contrast to that of the daily number of reported infected cases. These figures show that within each studied city, the numbers of “like”, “reply”, and “retweet” have a fairly similar trend, and their peaks appear around the middle of March 2020. Such peaks may be partly attributed to the peak of infected cases that is reported in early March, 2020 (Jiang et al. [31]).

**Fig 5:**
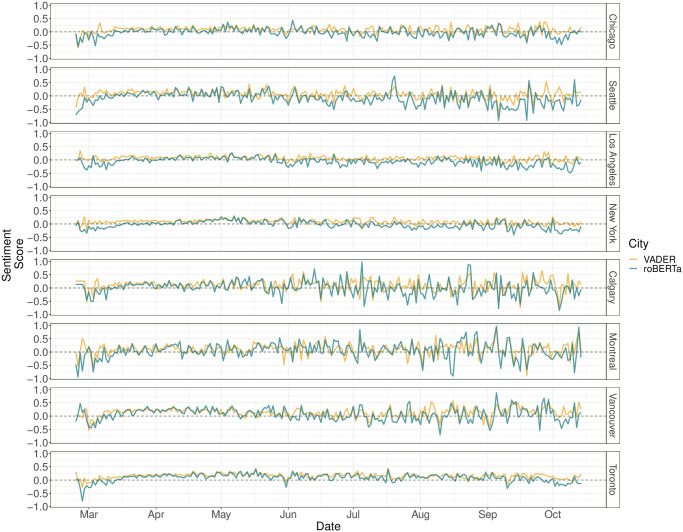
The line plot of the sentiment score of “COVID19” related tweets over time for the eight cities in North America, calculated using Vader lexicon and roBERTa, respectively.

Fig 5 presents line plots of the daily average sentiment scores for the eight cities over time, with orange and green color representing sentiments assessed using VADER and roBERTa, respectively. The sentiment change varies from time to time and from city to city. From the end of February to early March, sentiments in all studied cities, except for Vancouver, show overall negative patterns. Between the middle of March and early June, all the cities show positive sentiments though the degree varies. After June, sentiment reaction fluctuates noticeably from city to city, with all cities having varying negative patterns in September, which may be associated with a new surge of COVID-19 cases in September, as shown in the dynamic UWO website (https://covid-19-canada.uwo.ca/en/data.html) for Canadian data and the JHU website (https://coronavirus.jhu.edu/region/united-states) for the U.S. data. Overall, Toronto and New York remain fairly positive between the middle of March and the beginning of September, whereas other cities exhibit varying negative sentiments intermittently.

**Fig 6:**
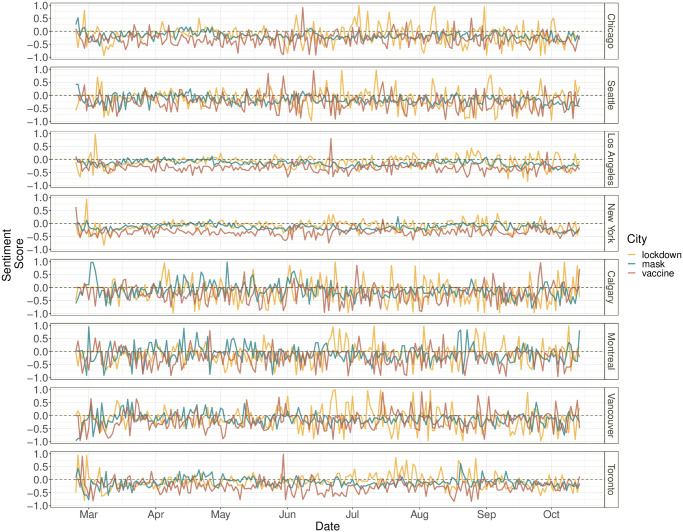
The line plots of the sentiment score of “mask”, “vaccine”, and “lockdown” related tweets calculated using roBERTa over time for the eight cities in North America.

To trace the change of sentiment scores in different cities over time, we summarize the mean and standard deviation of sentiment scores as well as the average daily number of tweets, stratified by city and the time periods shown in Fig 1, and we report the results in Table 6. For all the cities in the study, tweeting is the most active in Period 2 (i.e., the lockdown period) than other two periods, and the largest mean sentiment scores appear in Period 2 as well. Compared to Period 2, smaller mean scores in Periods 1 and 3 suggest more negative sentiments in the early stage of the pandemic and the re-opening period. The sentiment scores in the U.S. tend to be more negative or smaller than those in Canada in all the periods. While the mean scores differ in cities, the associated standard deviations remain similar for different cities and different periods.

**Table 6:** The average number of daily tweets (average #), together with the mean and standard deviation (s.d.) of tweetwise sentiment scores calculated from the RoBERTa method for different cities in different time periods.

		Canada				United States			
Period	Measure	Calgary	Montreal	Toronto	Vancouver	New York	Los Angeles	Seattle	Chicago
1	average #	25.26	7.29	95.78	36.58	47.23	12.90	8.33	13.07
	mean scores	0.01	-0.19	-0.01	0.00	-0.18	-0.17	-0.31	-0.18
	s.d.	0.58	0.50	0.58	0.60	0.58	0.59	0.58	0.52
2	average #	30.41	26.68	158.46	52.21	275.59	170.75	58.95	94.02
	mean scores	0.11	0.06	0.16	0.20	0.04	0.02	0.04	0.04
	s.d.	0.61	0.62	0.63	0.63	0.64	0.63	0.64	0.63
3	average #	5.89	6.85	47.39	10.51	75.27	48.19	13.18	22.57
	mean scores	-0.03	0.02	0.08	0.01	-0.09	-0.08	-0.11	-0.03
	s.d.	0.63	0.60	0.60	0.65	0.64	0.63	0.67	0.64

To see how anti-epidemic measures may be related to the daily average sentiment scores of the tweets obtained from the RoBERTa approach, we produce line plots for three keywords: “mask”, “lockdown”, and “vaccine”, and display them in Fig 6. Regarding opinions on “mask”, daily average sentiment scores are fairly close to zero for Toronto and the four U.S. cities, whereas they seem to fluctuate more noticeably for Vancouver, Montreal and Calgary, especially for early stages. In terms of opinions about “lockdown”, except in Los Angeles and New York, average sentiment scores for other cities exhibit seen variabilibility over time, especially for later periods. With regard to “vaccine”, average sentiment scores in Los Angeles and New York appear to be less variable with time than in other cities.

**Fig 7:**
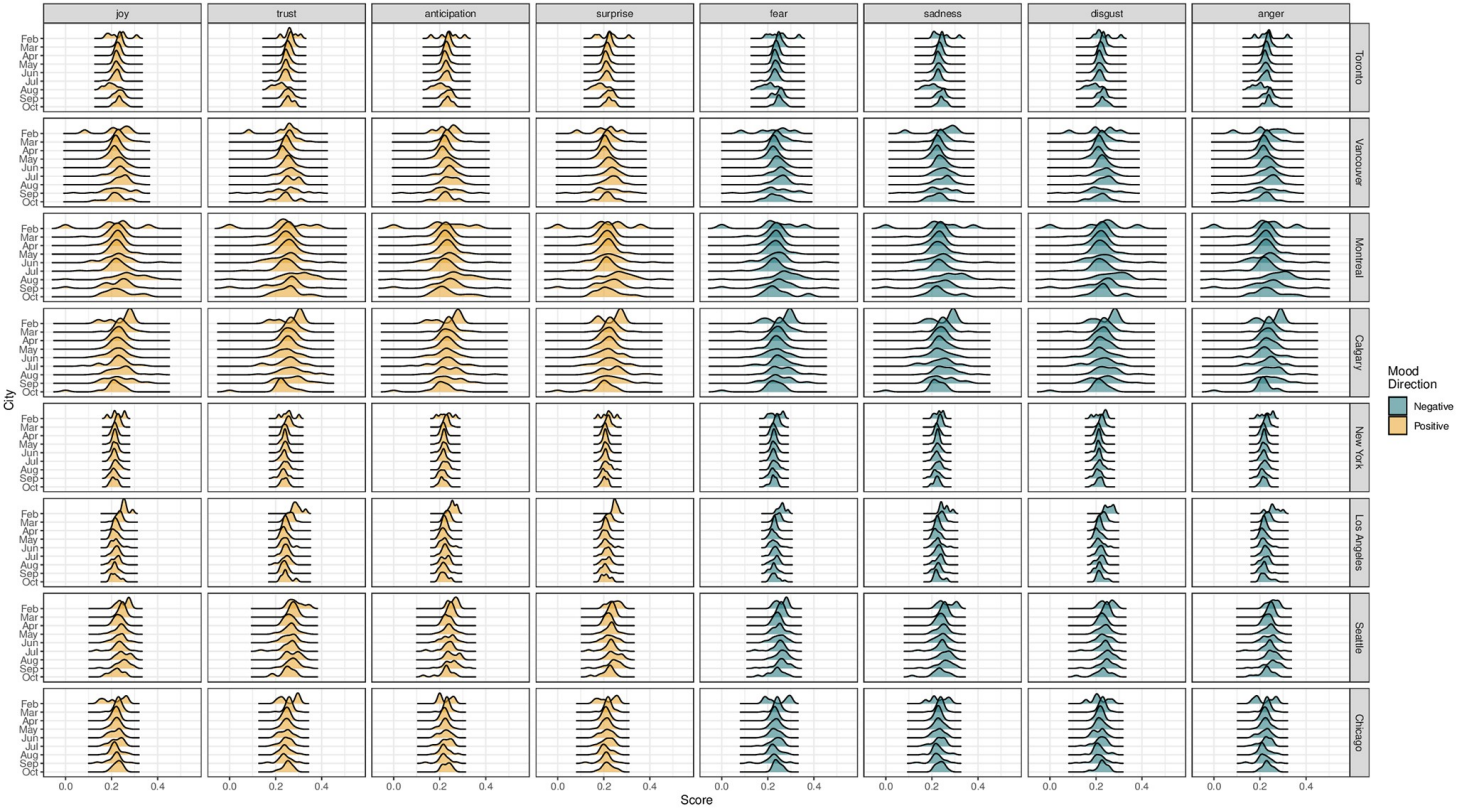
The density graph of the distribution of the moods frequency for different cities over 234 days.

To visualize the change in the mood composition of the daily tweets over time, in Fig 7, we present density plots obtained from the NRC method. Overall, the changes in Canadian cities seem to be more variable than in U.S. cities, and the trend and trajectory vary from city to city and from time to time. For example, from May to September, moods are slightly intensified in Vancouver, Montreal, and Calgary, and an opposite trend is observed for Toronto and New York. Relative to other months, February is the month that incurs a large variation for the word frequency in each mood for most cities.

### 4.2 Relationships between features

Now we pairwisely examine possible causal relationships by pairing five variables concerning COVID-19 tweets: RoBERTa sentiment scores, the daily number of tweets, the daily total number of “like”, the daily total number of “retweet”, and the daily total number of “reply”. Assuming that the causal relationships among those features are the same for each city, we merge the data in all cities and consider two directions of the relationship as explained in Section 3.2.1.

To illustrate the implementation of the procedures described in Sections 3.2, we consider a pair of variables, *X* and *Y*, to examine possibly two directional causal relationships, where for example, *X* represents the daily “like” counts of a city, and *Y* stands for the daily average sentiment scores in that city calculated from the RoBERTa method. Let {*X*_*t*_ : *t* = 1, …, *T*} and {*Y*_*t*_ : *t* = 1, …, *T*} denote the observed time series for *X* and *Y*, respectively. In Direction 1, we examine whether there is evidence to support *Y* to be the cause of *X*; and in Direction 2, we assess whether there is evidence to indicate *X* to be the cause of *Y*.

Applying the leave-one-out cross-validation in combination with the information in Table 5, we obtain that tuning parameters λ_max_, *N*, *p*_*s*_, *α*, and, *γ*, are, respectively set to be 0.1, 150, 0.1, 0.1, 0.9 in Direction 1, and 0.1, 250, 0.7, 100, 0.9 in Direction 2. Then using those parameter values, we train the echo state network for *W*_out_ to the time series *X*_*t*_ and *Y*_*t*_ to predict ${\hat{Y}}_{t}$ and ${\hat{X}}_{t}$ for both directions, respectively. The predictions are repeated for all the cities considered in the study. Finally, for each *τ* ≤ *T*, in Direction 1 we compute the Pearson correlation coefficient *ρ*_*y*_(*τ*) between ${\hat{Y}}_{t+\tau }$ and *Y*_*t*+*τ*_, and in Direction 2, we calculate the correlation coefficient *ρ*_*x*_(*τ*) between ${\hat{X}}_{t+\tau }$ and *X*_*t*+*τ*_, where ${\hat{Y}}_{t+\tau }$ is the predicted value corresponding to *Y*_*t*+*τ*_ in Direction 1 and ${\hat{X}}_{t+\tau }$ is the predicted value of *X*_*t*+*τ*_ in Direction 2.

**Fig 8:**
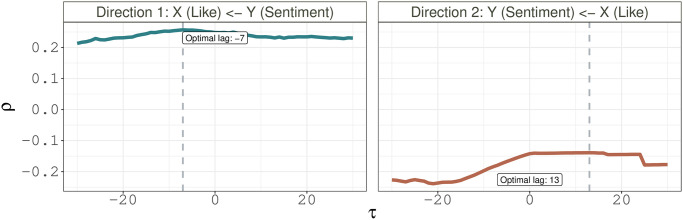
Pearson correlation coefficient versus different choices of lags for examining the possible causal relationship between *X* and *Y*, where *X* represents the daily like counts of a city, and *Y* stands for the daily average sentiment scores in that city calculated from RoBERTa method. Here Direction 1 examines whether *Y* is the cause of *X*, and Direction 2 assesses whether *X* is the cause of *Y*. The dash vertical line refers to the choice of lag that achieves the peak of the correlations.

We present the analysis results in Fig 8, which shows that in Direction 1, *ρ*_*y*_(*τ*) reaches the peak at *τ* = −7, whereas in Direction 2, *ρ*_*x*_(*τ*) is peaked at *τ* = 13 though it appears fairly flat around the peak. This suggests that the sentiment of COVID-19 is likely to cause the changes in the time series of “like” counts, but not vice versa.

**Fig 9:**
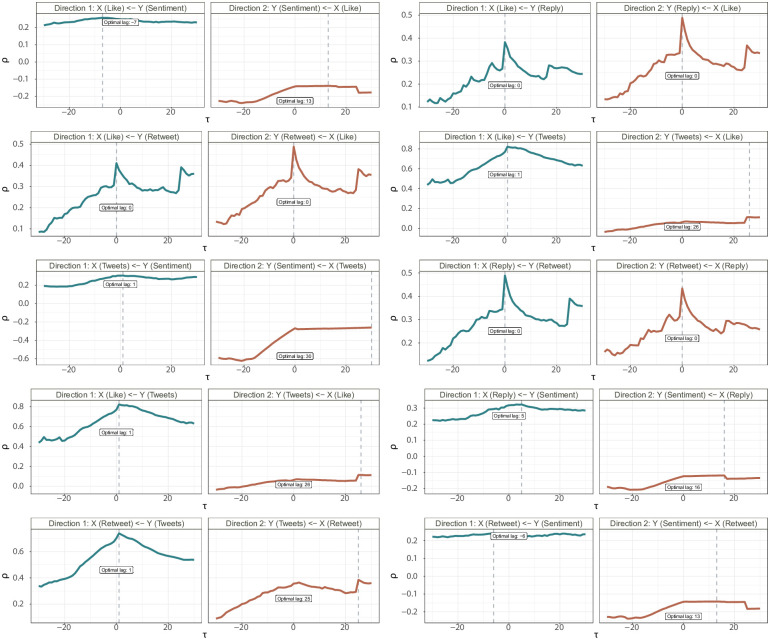
Pearson correlation coefficient versus different values of *τ* for examining the possible causal relationship between *X* and *Y*, taken from a pair of variables among sentiment scores calculated using RoBERTa, the daily number of tweets, the daily number of likes, daily number of reply, and the daily number of retweets. Here Direction 1 examines whether *Y* is the cause of *X*, and Direction 2 assesses whether *X* is the cause of *Y*. The dash vertical line refers to the choice of lag that achieves the peak of the correlations.

**Fig 10:**
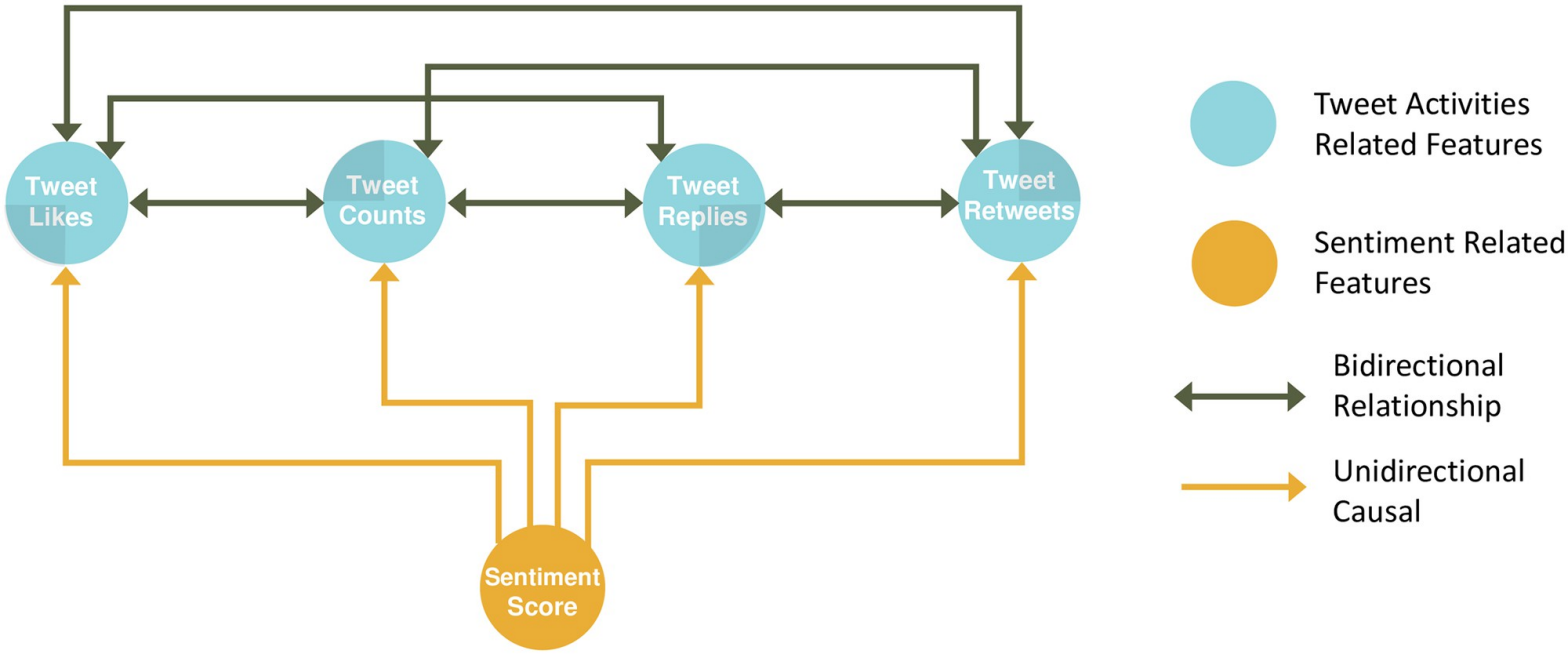
Summary of causal relationships for the variables associated with the tweet activities and sentiment scores.

Repeating the preceding analysis to each remaining pair of five variables about COVID-19 tweets, in Fig 9 we report plots of Pearson correlations versus the *τ* values. The results reveal: (i) the sentiment scores unidirectionally cause the changes in the tweet-related features, including “tweet”, “like”, “reply”, and “retweet” counts, but not vice versa; and (ii) the tweet activity features of “tweet”, “like”, “reply”, and “retweet” are bidirectionally related to each other. The results of the causal relationships are summarized in Fig 10, whose validity relies on an implicit assumption of no existence of confounders when examining the causal relationship for any paired variables.

## 5 Conclusion and discussion

The COVID-19 pandemic has presented tremendous challenges to the public. It is interesting to study public sentiments in reacting to anti-epidemic measures. In this paper, we conduct a sentiment analysis of the tweets of “COVID-19” as well as topics of “masks”, “lockdown”, and “vaccines” prior to the rollout of COVID-19 vaccines, collected in eight North American cities. We apply the echo state network and convergent cross-mapping to examine possible causal relationships between paired variables concerning COVID-19 tweets. The study shows that public sentiments change over time, differ from city to city, and vary with respect to the anti-epidemic measures of “lockdown”, “vaccine” and “mask”. Under the assumed model, there is evidence suggesting that sentiment scores may be the unidirectional cause of the changes in the tweet-related features, such as “tweet”, “like”, “reply”, and “retweet” counts, and that the tweet activity features are bidirectional correlated with each other. The influence of sentiment scores on people’s behavior in tweeting about COVID-19 is consistent with the revealings by others. For example, Stieglitz and Dang-Xuan [32] found that the tweets with more intensive emotions tend to be retweeted more frequently and faster than others.

While our analyses provide insights into understanding people’s sentiments and emotions about *COVID*−19, several limitations need to be considered. The analyses are conducted to the gross number of tweets in each study region, with the size differences in cities ignored. Our study is directed to the tweets about COVID-19, which are small in size relative to the large volume of tweets on other topics. With more tweets about COVID-19 becoming available as the pandemic evolves, it can be interesting to apply normalization or transformation to account for varying sizes of regions. One may also delve into further sentiment analysis to capitalize on the advantage of social media over traditional survey schemes, and do comparisons between COVID-19 related and non-related tweets.

An important issue concerns the representativeness of the tweets we analyze. As the available tweets come from those Twitter users, our study is basically carried out for observational data rather than data arising from a random sample. The analysis here is conducted on English tweets. Tweets not in English, such as French tweets, primarily coming from Montreal, are not included in the study. The results here thereby cannot truthfully reflect the opinions of the entire population. The interpretation of the results must be done for a subpopulation of English Twitter users instead of the whole population; otherwise, biased conclusions are expected. Further, the information of tweets is not fully used. For example, figure memes and the voice records in the tweets are not utilized.

The analyses here are carried out at the city level for tweets coming from the United States and Canada. Taking “city” as a unit to group tweets helps acquire a reasonable size of data on a daily basis. The analyses thereby basically ignore the variation of sentiments within cities. When richer sources of data become available, it is interesting to investigate how differently setting a study unit may affect analysis results and how variability within units may be accounted for in the analysis.

We study tweets about COVID-19 for the largest cities (Toronto, Montreal, Vancouver, Calgary) in the four most populated Canadian provinces and the three most populated cities (New York, Los Angeles, Chicago) in the United States, plus Seattle which is in the vicinity of Vancouver. It is interesting to extend the study here to more cities to compare how sentiments about COVID-19 may be associated with different cities of small sizes.

Our analyses root in the data collected prior to the rollout of the COVID-19 vaccines, and this is primarily driven by the interest in conducting sentiment analysis for the pre-vaccines period. We perceive that sentiment patterns may be considerably different before and after the advent of vaccines. It is interesting to develop a real-time and interactive platform (e.g., [33]) to reveal dynamic changes in sentiments over time. It is useful to examine how sentiments may be influenced by major associated factors, such as the advent of vaccines, the availability of easily accessed rapid tests, changes in preventative policies, emerging of new virus variants, and so on.
